# Kommentar zu: Arbeits(zeit)schutz nach Art der BRD – am Beispiel der COVID-19-Arbeitszeitverordnung

**DOI:** 10.1007/s41449-021-00261-y

**Published:** 2021-07-01

**Authors:** Florian Steger

**Affiliations:** Institute für Geschichte, Theorie und Ethik der Medizin, Universität Ulm, Parkstraße 11, 89073 Ulm, Deutschland

Keiner dürfte ernsthaft Zweifel an der Bedeutung des Arbeitsschutzes anmelden. Vielmehr stellt das hohe Arbeitsschutzniveau ein großes Verdienst dar, für das in der Geschichte auch sehr lange Zeit gestritten und gekämpft wurde. Insofern ist es mehr als verständlich, wenn Schwächungen oder zumindest zeitweise Aussetzungen dieses hohen Arbeitsschutzstandards zu Kritik führen.

Eben diese Kritik wird in dem vorliegenden Beitrag „Arbeits(zeit)schutz nach Art der BRD – am Beispiel der Covid-19-ArbZV“ geübt. Im Detail geht es um die vom BMAS mit BMG im April 2020 erlassene Covid-19-ArbZV, die vom 10.04.2020 bis 30.06.2020 mit Folgen bis 31.07.2020 wirkte. Ziel der Verordnung war die Sicherstellung der Versorgung von Patienten und der allgemeinen Bevölkerung. Hierfür wurden erhebliche Abweichungen vom ArbZG ermöglicht, die zu einer Senkung des Arbeitsschutzniveaus führe und damit zu einer Erhöhung der Risiken für die Beschäftigen selbst, aber auch für die Patienten und die allgemeine Bevölkerung. Der Sorge, für die Sicherstellung der Patientenversorgung und der allgemeinen Bevölkerung zu wenig Personal zur Verfügung haben, stand das erhöhte Risiko für Sicherheit und Gesundheit der Beschäftigten und der Patienten bzw. der allgemeinen Bevölkerung gegenüber.

Der individuelle Schutz des einzelnen aber auch der Öffentlichkeit würde, so im Beitrag, durch die Covid-19-ArbZV also mehr gefährdet als hierdurch gewahrt werden. Die beschlossenen Änderungen von Standards, denen historische Aushandlungsprozesse zugrunde liegen, könnten also zu erheblichen Folgen führen – so handle es sich um exponentielle Zusammenhänge mit überproportionalen Effektsteigerungen, die den einzelnen aber auch die Gesellschaft betreffen. Eine Schwächung des Arbeitsschutzes führe letztlich zu erheblichen Konsequenzen für den einzelnen, aber auch für die Gesellschaft. So stehe die Gesundheit des Beschäftigten, dessen soziale Interaktion mit seinem unmittelbaren Umfeld (soziale Teilhabe), aber auch die Gesundheit des zu versorgenden Patienten durch erhöhte Fehlerwahrscheinlichkeit bei der Patientenversorgung auf dem Spiel; letztlich könnten die beschlossenen Änderungen des Arbeitsschutzniveaus also zu erheblichen gesellschaftlichen Beeinträchtigungen führen.

Diese kritischen Überlegungen werden fundiert und überzeugend deskriptiv vorgetragen und erscheinen mir auch prima facie plausibel. Zugleich befinden wir uns in einer globalen Pandemie, die uns in kürzester Zeit in eine Ausnahmesituation gebracht hat. Oberstes Ziel muss es sein, das Wohl des einzelnen gleichermaßen als auch das der Öffentlichkeit zu wahren. Entsprechend engagiert sind die Bemühungen, die Pandemie zu begrenzen und den hieraus entstehenden Schaden so gering wie möglich zu halten. Als Zeitzeugen dieser Pandemie haben wir gelernt, wie dünn die Wissensbasis ist, und wie schwierig es ist, wissenschaftlich belastbare Aussagen zu treffen. Wir sind täglich damit konfrontiert, mit dieser epistemischen Unsicherheit umzugehen. Und dennoch müssen letztlich politische Entscheidungen getroffen werden, die bei aller Unsicherheit legitimierte Mandatsträger zu verantworten haben. Politische Beschlüsse in der Pandemie herbeizuführen wird letztlich nicht ohne Fehlerhaftigkeit möglich sein. Umso wichtiger ist es dann, auf der Basis des verfügbaren Wissens unter Einbezug hoher Expertise zu wohlerwogenen Entscheidungen zu kommen, welche allerdings durchaus revidiert werden können und – wie wir in der Pandemie immer wieder sehen – auch müssen. Gerade das hohe Tempo der Pandemie führt auch dazu, dass die Zeit zu kurz ist, um belastbare empirische Ergebnisse zum Beispiel in einer klinischen Studie zu gewinnen, auf deren Basis dann eine belastbare Politikberatung erfolgen könnte. So wird beraten, diskutiert und empfohlen, aber die Wissensbasis der Entscheidung ist und bleibt nun einmal dünn. Der politische Beschluss wird durch das Mandat legitimiert.

In dem vorliegenden Beitrag wird ein düsteres Bild entworfen, welche Konsequenzen die Senkung des Arbeitsschutzniveaus habe; dabei steht die Covid-19-ArbZV in der Kritik. Um nicht missverstanden zu werden, ich finde selbst die Errungenschaften, welche wir durch den Arbeitsschutz erreicht haben, enorm, und ich würdige dies vor allem auch in Anbetracht der hierfür notwendigen gewesenen historisch sehr lange dauernden Aushandlungsprozesse. Ich finde es plausibel, dass eine Ausdehnung der Arbeitszeit am Tag und in der Woche sowie die Reduktion der Ruhezeit am Tag und in der Woche zu erheblichen gesundheitlichen Konsequenzen für den Beschäftigen und auch zu Beeinträchtigungen seiner sozialen Teilhabe führen können, es scheint mir auch plausibel, dass bei solcher Belastung das Risiko für die Patienten bei dessen unmittelbaren Versorgung steigt. Und das alledem letztlich überproportionale Wirkzusammenhänge zugrunde liegen, wiegt schwer und macht mich besorgt. Dies könnte auch erhebliche Konsequenzen für unsere gesellschaftliches Zusammenleben haben. Zugleich gilt es aber auch die Ausnahmesituation zu würdigen, in welcher diese Veränderung politisch beschlossen wurde. Es ist das enorme Tempo der Pandemie und an sich noch mehr das hohe Tempo der politischen Beschlüsse sowie die epistemische Unsicherheit, die solchen wohlüberlegten Entscheidungen zugrunde liegt, vor Augen zu führen.

In dem vorliegenden Beitrag wird modelliert, um Risikoabschätzungen vorzunehmen. Es werden Risikobeurteilungen vorgenommen, die letztlich Trends abbilden. Und sicherlich wird hier auch etwas dran sein, es ist völlig nachvollziehbar, dass bei einer Absenkung des Arbeitsschutzniveaus das Risiko der Gefährdung von Gesundheit, sozialer Teilhabe und Sicherheit erhöht ist. Zugleich wünscht man sich an dieser Stelle aber klinische Studien, die eben diese Beeinträchtigungen repräsentativ nachgewiesen haben. Und in Ermangelung der Möglichkeit, solche Daten jetzt unmittelbar vorzulegen, wird modelliert. Das Tempo bestimmt also wieder einmal die Belastbarkeit der Aussage. Allerdings könnte man durchaus die Beeinträchtigungen durch die Covid-19-ArbZV retrospektiv erfassen und hätte aus meiner Sicht eine bessere argumentative Grundlage als jetzt, wenn man anhand einer Modellierung Risiken abschätzt und Trends nach Plausibilität betrachtet. Ja, die Modellierungsergebnisse sind beeindruckend, nahezu erschreckend, wenn man sich das deutlich erhöhte Risiko betrachtet, welche durch solche Änderungen im Arbeitsschutzniveau entstehen können. Kritisch könnte man spätestens an dieser Stelle einwenden, ob dieses Arbeitsschutzniveau in der Gesundheitsversorgung außerhalb der Pandemie tatsächlich bestand. Natürlich bestanden diese Normen, und sicherlich wird man auch sehr bemüht gewesen sein, dieses bei der täglichen Arbeit umzusetzen. Allerdings frage ich mich, was wirklich an der Basis von diesem hohen Arbeitsschutzniveau auch gelebt wurde, blickt man in die Gesundheitsversorgungssysteme hinein. Das soll kein Gegenargument sein, vielmehr einmal mehr deutlich machen, dass wenn es um solches Gefährdungspotential der Beschäftigten aber auch der Patienten geht, empirische Daten der gelebten Realität von größter Bedeutung sind. Die Covid-19-ArbZV wurde beschlossen, um die Patientenversorgung und die der allgemeinen Bevölkerung sicherzustellen. Wenn man hierfür eine erhebliche Gefährdung der Beschäftigten und der Patienten sowie letztlich der Gesellschaft riskiert, ist dringend eine kritische Diskussion und Evaluierung der Verhältnisse geboten. Gravierend ist hierbei nicht zuletzt, dass in unserem Zusammenhang dem Risiko für Fehlhandlungen im Gesundheitssystem exponentielle Wirkungen zugrunde liegen. Wenn also die Covid-19-ArbZV letztlich zu einer Absenkung des Niveaus der Patientenversorgung, vielmehr zu einer gesundheitlichen Gefährdung der Beschäftigten und der Patienten sowie darüber hinaus der Gesellschaft führt, gehört die Covid-19-ArbZV einmal mehr auf den kritischen Prüfstand.

In dem Beitrag wird beeindruckend gezeigt, welche arbeitswissenschaftliche Expertise vorhanden ist, die unbedingt in einen politischen Entscheidungsprozess eingebunden werden muss. Und zugleich sind die Limitationen der Aussagen auch dieses Beitrags zu bekennen, dass bei aller Nachvollziehbarkeit und Plausibilität, letztlich auch hier Modellrechnungen für Risikobeurteilungen zugrunde liegen. Das macht aber nicht wirklich etwas, wenn es explizit ist. Sehr viele Entscheidungen, die in der Pandemie getroffen wurden, haben keinen empirisch belastbaren Wissensstand. Eben diesem Dilemma der epistemischen Unsicherheit kann man dann nur durch die kritische Diskussion unter Einbeziehung möglichst großer und vielstimmiger Expertise begegnen, bei der, das zeigt der Beitrag eindrucksvoll, auch die arbeitswissenschaftliche Expertise ihren angemessenen Platz verdient. Allerdings wird es in einer Pandemie am Ende zu einer politischen Entscheidung kommen müssen, die letztlich durch die Mandatsträger demokratisch legitimiert und zu verantworten ist.

Prof. Dr. Florian Steger (Ulm)

